# Utilization of MANAGE-PD Tool in a Real-World Setting in Germany: A Cross-Sectional Study

**DOI:** 10.3390/brainsci14070630

**Published:** 2024-06-24

**Authors:** Martin Südmeyer, David J. Pedrosa, Frank Siebecker, Carolin Arlt, Jaakko J. Kopra, Wolfgang H. Jost

**Affiliations:** 1 Department of Neurology, Ernst-von-Bergmann Klinikum, 14467 Potsdam, Germany; 2 Department of Neurology, Medical Faculty, University Düsseldorf, 40225 Düsseldorf, Germany; 3 Department of Neurology, Philipps-University Marburg, 35037 Marburg, Germany; pedrosac@staff.uni-marburg.de; 4 Praxis Neurologie, 48291 Telgte, Germany; fs@neurologie-telgte.de; 5 AbbVie GmbH, 1230 Wien, Austria; carolin.arlt@abbvie.com; 6 AbbVie Deutschland GmbH & Co. KG, 65189 Wiesbaden, Germany; jaakko.kopra@abbvie.com; 7 Parkinson-Klinik Ortenau, 77709 Wolfach, Germany; w.jost@parkinson-klinik.de

**Keywords:** Parkinson’s disease, MANAGE-PD, clinical decision-making, physician-patient concordance, device-aided therapy, patient identification

## Abstract

MANAGE-PD is a validated, web-based tool to assist physicians in identifying patients with Parkinson’s disease (PD) whose symptoms are inadequately controlled by oral medication. Also, a modified patient version of MANAGE-PD (Parkinson Check) is available in Germany. However, prospective research into the clinical utility of MANAGE-PD is lacking. This non-interventional study aimed to assess the real-world clinical utility of the MANAGE-PD and Parkinson Check in PD patients attending a single visit at specialist clinics and neurologist practices in Germany in 2022. Participants’ disease control was rated by the physicians using their own judgment, and by completing the MANAGE-PD, and by the patients completing the Parkinson Check. Concordance was calculated between the unassisted physician’s assessment and the outcome of MANAGE-PD, as well as the Parkinson Check. A total of 278 patients from 19 sites were included in the analyses, of whom 160 patients (57.6%) were assigned to the same category of disease control by physicians’ judgment and the MANAGE-PD. Concordance was higher in patients treated in specialist clinics (63.9%) than in neurologist practices (43.7%). Concordance between physicians’ and patients’ responses was high (>80%) for each question in the Parkinson Check. MANAGE-PD proved to be especially valuable for general neurologists in identifying patients who should be referred to specialist clinics. The Parkinson Check self-assessment generated promising outcomes that merit its more widespread use.

## 1. Introduction

The main pillar for the treatment of Parkinson’s disease (PD) has remained largely unchanged for many years and still consists of dopaminergic medication. However, symptom control based on dopaminergic substitution becomes less effective after a few years of treatment due to the narrowing of the therapeutic window and treatment-related side effects, as well as erratic gastric emptying as PD progresses [1]. 

Since there is no clear definition of when a patient has reached advanced PD or inadequate symptom control with oral medications [2,3,4,5,6,7], there is a lack of clinical data describing who might benefit from further assessment to optimize current medication and/or from adding device-aided therapy (DAT). In addition, symptoms may be perceived and assessed differently by physicians and patients. As a result, there may be inconsistencies in the decision-making process and delayed referral and assessment for a DAT option [8,9,10,11]. The currently available DAT treatment options include deep brain stimulation (DBS), levodopa–carbidopa intestinal gel (LCIG) infusion, levodopa–entacapone–carbidopa intestinal gel (LECIG) infusion, continuous subcutaneous apomorphine infusion (CSAI), and, most recently, subcutaneous foslevodopa/foscarbidopa infusion (LDp/CDp) [12,13].

To address these difficulties, criteria for advanced PD and eligibility for DATs were developed by leading experts in the field using the Delphi method [14]. Based on the results of this Delphi consensus, the Making Informed Decisions to Aid Timely Management of Parkinson’s Disease (MANAGE-PD) tool was created. The MANAGE-PD aims to assist physicians in identifying patients with PD whose symptoms are not well controlled on oral medication and who may be eligible for device-aided therapies by assigning each patient to one of three categories of disease control [15]. The MANAGE-PD tool has been validated [15] and registered as a class 1 medical device. It is available for free online (www.managepd.eu, accessed on 18 June 2024) and as a paper-based version in Germany since 2020. However, prospective research into the clinical utility of MANAGE-PD under real-world conditions has not been reported.

The Parkinson Check (patient self-assessment version of the MANAGE-PD tool Section 1) was developed in Germany to support patients in assessing their level of symptom control on oral therapies and to communicate this information to their treating physician. It was made available online in Germany in 2021 (www.abbvie-care.de/parkinson-check/selbsttest, accessed on 18 June 2024). In this study, a paper-based version of the Parkinson Check was collected as a patient-reported outcome measure (PROM) for the first time. In many countries outside Germany, a similar patient version called “MY PD-CARE” (www.mypdcare.com, accessed on 18 June 2024) is available.

This study attempted to address three objectives: First, the data should elucidate for the first time how reliably the Parkinson Check could be used by patients and whether this self-assessment may hold potential for routine clinical use. Second, differences in outcomes between defined patient subgroups (including sex, age, and time since diagnosis), as well as physician characteristics (specialist clinic and neurologist practice), were assessed. Third, the output of the tool was meant to be compared with the unassisted physician’s judgment in this patient cohort to complement the original validation study [15] with prospective data.

## 2. Materials and Methods

### 2.1. Study Design and Study Population

In this observational, cross-sectional, non-interventional multicenter study, adult patients diagnosed with idiopathic PD (according to the UK Parkinson’s Disease Society Brain Bank criteria) were enrolled at specialist clinics (*n* = 10) or neurologist’s practices (*n* = 9) in Germany between January and September 2022. Only PD patients with current oral levodopa therapy but without current or past treatment with a DAT could be included. All participating physicians were experienced in the management and treatment of patients with PD, were trained for use of the MANAGE-PD tool prior to the study start, and conducted the study in accordance with applicable legal and regulatory requirements. The study was conducted in accordance with the latest version of the Declaration of Helsinki and was approved by the ethics committee of Landesärztekammer Brandenburg (No. 2021-2188-BO-ff). All participating patients gave written informed consent.

### 2.2. Data Collection

Demographic and clinical characteristics were collected from each patient at a single routine visit. Patients’ disease control was first rated by the physician and then using the MANAGE-PD tool [15]. Details on the classification process of the MANAGE-PD tool are illustrated in Supplemental Figure S1. The tool comprises two Sections: Section 1 includes five questions on the assessment of inadequate disease control by current oral treatment. If all questions in Section 1 are answered with “no” (including fewer than 5 levodopa doses per day), the tool’s algorithm classifies the patient into Category 1 (“adequately controlled on current oral therapy”). If any of the questions in Section 1 are answered with “yes”, the patient is assessed as inadequately controlled, and the tool proceeds with Section 2. In Section 2, based on ten additional questions, the patient’s eligibility for DAT is determined. In detail, the physicians assess frequency and severity of motor symptoms, non-motor symptoms, and functional limitations. The tool’s algorithm assesses whether the severity and frequency of any domain is higher than an established threshold and classifies the patient into either Category 2 (“inadequately controlled under current therapy—might benefit from further non-DAT optimization”) or Category 3 (“inadequately controlled under current therapy—might benefit from DAT”) [15,16]. In this study, participants were also asked to complete Section 1 of the patient self-assessment version of the MANAGE-PD tool in a paper-based version to allow for comparisons with the physician’s assessment.

Usability and benefit of the MANAGE-PD tool (AbbVie Deutschland GmbH & Co. KG, 67061 Ludwigshafen, Germany) were rated by the physicians on a scale ranging from 0 (very bad) to 10 (very good). In the same way, the usability of the self-assessment version of MANAGE-PD was rated by the patients.

### 2.3. Statistical Analysis

All data were analyzed descriptively in all patients who fulfilled selection criteria and were stratified by state of disease control according to the MANAGE-PD tool (categories 1–3). Concordance between the physician’s assessment (without using MANAGE-PD tool) and the outcome of the MANAGE-PD tool was calculated as the percentage of patients that were assigned to the same category by the physician and by the tool. The concordance between physicians and patients regarding the five questions of Parkinson Check was calculated in the same way. Additionally, subgroup analyses were performed by the median patients’ age (≤72 years, >72 years), sex (male, female), institution type (specialist clinic, neurologist practice), and time since diagnosis (<10 years, ≥10 years). The cut-off of 10 years was chosen, as it has been shown that after 10 years of PD, the majority of patients exhibit non-motor symptoms [17].

## 3. Results

### 3.1. Patient and Site Characteristics

In total, 287 patients were enrolled at 19 sites. A total of 278 patients (96.9%) fulfilled the selection criteria and were included in the analyses. The mean age was 70.8 years, and almost two-thirds of patients were male (Table 1). On average, the time since diagnosis was 7.4 years. In total, 10 specialist clinics enrolled 191 patients, and 9 office-based neurologist practices enrolled 87 patients. In comparison to patients in Category 2 and 3, patients in Category 1 showed the shortest mean time since diagnosis and time since first PD symptoms, as well as the lowest frequency of patients with advanced state of disease and care-giver support.

### 3.2. Disease Control According to the MANAGE-PD Tool and Physician’s Judgment

**Figure 1:**
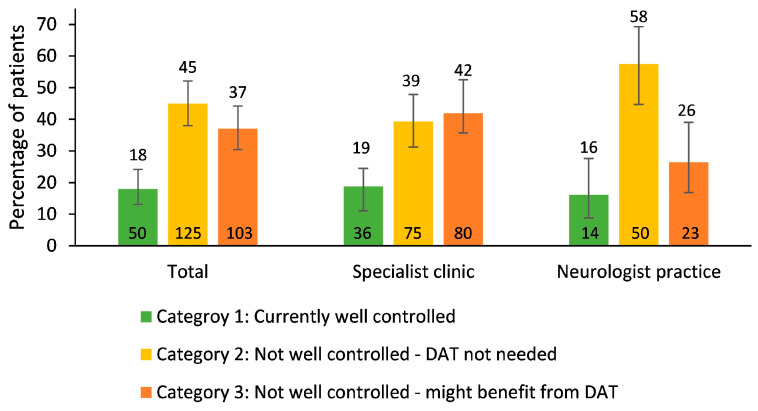
Disease control according to MANAGE-PD tool (percentages of patients together with 95% confidence intervals). The number of patients in each category is given at the bottom of the bars. DAT, device-aided therapy.

According to the MANAGE-PD tool, 50 patients (18%) were currently well controlled (Category 1), 125 patients (45%) were not well controlled but might not yet need a DAT (Category 2), and 103 patients (37%) were not well controlled and might benefit from DAT (Category 3) (Figure 1). The percentage of patients in Category 3 was higher for patients treated in specialist clinics (42%) than for patients treated in neurologist practices (26%). The corresponding numbers, according to unassisted physician’s judgment, were 74 patients (26.6%) in Category 1, 133 patients (47.8%) in Category 2, and 71 patients (25.5%) in Category 3.

**Figure 2:**
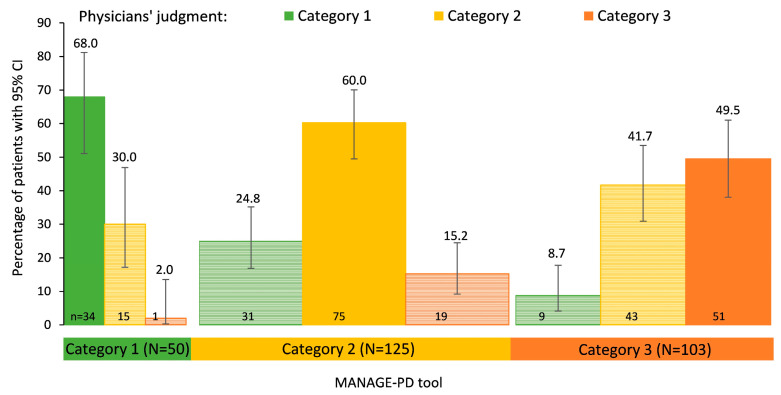
Physicians’ judgment of disease control by categories according to MANAGE-PD tool. Lighter bars denote a deviation between physicians’ judgment and the MANAGE-PD tool. The width of the bars represents the relative number of patients in each tool category.

Comparing the MANAGE-PD tool with physician’s judgment, 68% of patients in Category 1 (MANAGE-PD tool) and 60% of patients in Category 2 (MANAGE-PD tool) were assigned to the same category by the physicians, respectively (Figure 2). However, physicians rated almost one-fourth of the patients in Category 2 (MANAGE-PD tool) as well controlled. Concordance between the MANAGE-PD tool and the physicians was lowest for patients in Category 3 (MANAGE-PD tool): only half of the patients in this category were also assigned to Category 3 by the physicians. Hence, better disease control was assumed by the physicians in 50.5% of patients. Across all categories, 160 patients (57.6%) were assigned to the same category by the physicians and the MANAGE-PD tool.

**Figure 3:**
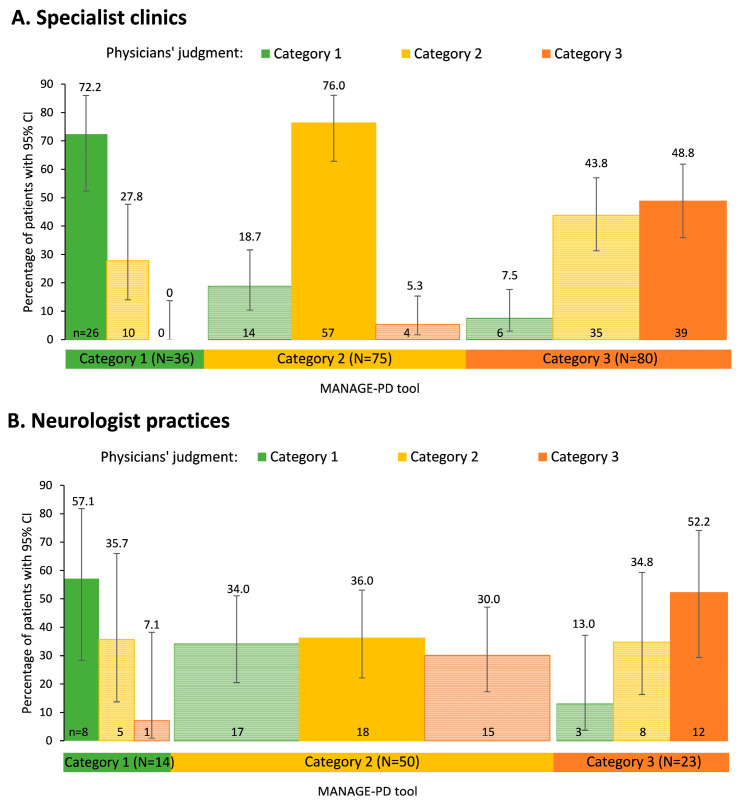
Physicians’ judgment of disease control by categories according to the MANAGE-PD tool in patients (**A**) treated in specialist clinics and (**B**) neurologist practices. Lighter bars denote a deviation between physicians’ judgment and the MANAGE-PD tool. The width of the bars represents the relative number of patients in each tool category.

Overall, concordance between physician’s judgment and the MANAGE-PD tool was higher in patients treated in specialist clinics (63.9%, Figure 3A) than for patients treated in neurologist practices (43.7%, Figure 3B). In particular, patients in Category 2 (MANAGE-PD tool) were assigned more often to the same category by physicians from specialist clinics (76.0%) than by physicians in neurologist practices (36.0%). The specialist clinics performed well compared with the tool in assigning patients to Categories 1 and 2 (over 70% concordance) but performed remarkably poorer in Category 3, where the concordance was only 48.8%.

Stratified analyses by age, sex, and time since diagnosis did not show essential or unexpected differences between the respective subgroups (data not shown).

### 3.3. Concordance between Physicians and Patients

**Figure 4:**
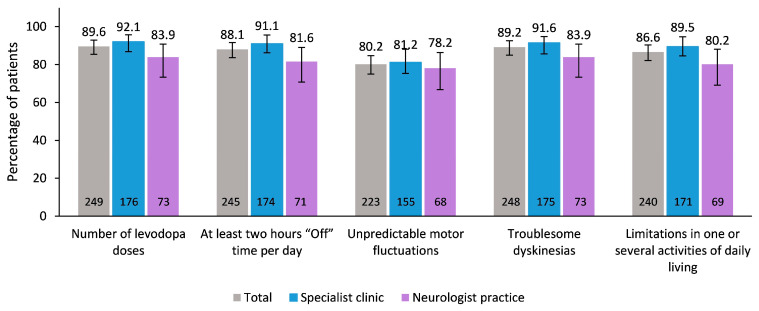
Concordance between physicians and patients (percentages of patients together with 95% confidence intervals). The number of patients is given at the bottom of the bars.

Overall, concordance between physicians’ and patients’ responses was high for each question of the Parkinson Check/MANAGE-PD tool Section 1 (Figure 4). The highest concordance was observed for Number of levodopa doses (89.6%) and Troublesome dyskinesia (89.2%). Unpredictable motor fluctuations showed the lowest concordance between physicians and patients (80.2%). In general, concordance between patients and physicians was higher in patients treated in specialist clinics than in patients treated in neurologist practices. The description of patients’ response is given in the Supplemental Table S1.

Stratified analyses by age, sex, and time since diagnosis did not show essential or unexpected differences between the respective subgroups (data not shown).

### 3.4. Tool Acceptance

On average, physicians rated the usability of the MANAGE-PD tool at 8.1 (out of 10) and its benefit at 6.0 (out of 10, Table 2). The mean usability was rated slightly higher by physicians from specialist clinics compared with physicians from neurologist practices. In contrast, the mean benefit rating was higher for physicians from neurologist practices than for physicians from specialist clinics. Overall, the mean time to complete Section 1 and Section 2 was 6 min for each section. The mean times to complete the sections by the physicians were higher for patients treated in neurologist practices than in specialist clinics. On average, patients rated the usability at 7.0 and needed 6.1 min to complete Section 1.

## 4. Discussion

The assessment of the MANAGE-PD tool seemed to reveal a clear insufficiency in the management of PD patients, as most of the study patients (82%) were not well controlled under their current oral therapy. Even though the study cohort may not be entirely comparable to the typical German PD patient population, this observation highlights the benefit of utilizing tools in order to identify suboptimally controlled patients.

This study also presents data for the first time on the validity, usability, and benefit of the Parkinson Check self-assessment (patient version of MANAGE-PD Section 1). Notably, with consistencies of 85% for most questions, there was high concordance in Section 1 responses by physicians and patients, i.e., in the assessment of inadequate oral treatment (MANAGE-PD Section 1). Hence, the data provide the basis for consideration of the future use of the patient self-assessment version of MANAGE-PD (Parkinson Check in Germany; MY PD-CARE outside Germany) in routine care, as it is easy to use and may thus be a feasible preparatory step for the patient’s next appointment. As a welcome secondary benefit, its use may increase patient engagement, as involving patients in the treatment evaluation could improve self-perception of their disease status by being better prepared and informed. Eventually, a more engaged patient could also facilitate the doctor’s visit, as the physician may not even need to fill in Section 1 anymore, or they may only be obliged to do so in selected cases in order to compare their assessment with one of the patients.

The concordance between the physician’s and patient’s responses to Section 1 was considerably affected by the institution type, as concordance between physicians’ and patients’ responses was higher among movement disorder specialists. Similarly, there was also a higher concordance between the MANAGE-PD tool and the physicians’ judgment in patients treated in specialist clinics than in neurologist practices. The most plausible explanation is that specialized training and thorough experience in the management of Parkinson’s disease allow movement disorder specialists to make more accurate and adequate diagnoses. Accordingly, one may conclude that by utilizing the MANAGE-PD tool, which was developed by leading international PD experts, general neurologists are granted the opportunity to align their decision with that of movement disorder specialists. However, many specialists may also benefit from the use of a more objective tool. This is evidenced by the fact that even in specialist clinics, considerably more patients were placed in Category 3 by the tool (42.0%) than according to the physicians’ judgment (25.5%). Moreover, the tool could improve the interface between general neurology practices and movement disorder clinics in the initiation of DATs. For example, a general neurologist could implement the initial oral therapy optimization (such as polypharmacy or dose fractioning) suggested for Category 2 patients and then refer the patient to a specialist clinic if the oral optimization does not provide an adequate level of control.

The MANAGE-PD tool was comprehensively evaluated and showed robust validity using both vignette-based and patient-based approaches, as reported by Antonini et al. [15]. Comparing the outcome of the validated MANAGE-PD tool and the unassisted physicians’ assessment of patients’ disease control, a moderate concordance was observed in the current study. Notably, compared with the MANAGE-PD tool outcome, physicians placed almost 50% more (74 versus 50) patients in Category 1 and over 30% less patients in Category 3 (71 versus 103). Thus, compared with the tool, physicians tended to overestimate the level of control on oral therapy. Therefore, MANAGE-PD may contribute to optimized disease control in these patients. Our concordance results are also supported by the real-world validation assessment published by Antonini et al., which was based on 1180 patients from a syndicated data source [15]. In that study, there was a concordance between the MANAGE-PD tool and clinical judgment in 55% of the patients, which is a very similar outcome to the 57.6% in our prospective study.

Other approaches, in addition to MANAGE-PD, have also been developed to screen/identify inadequately controlled PD patients [18]. The CDEPA questionnaire (Cuestionario De Enfermedad de Parkinson Avanzada (Questionnaire for Advanced PD)), developed in Spain using Delphi methodology, identifies advanced PD primarily based on “definitive symptoms” whose presence, even when isolated, classifies the PD patient as advanced [4,6]. These definitive symptoms include severe disability, motor fluctuations with limitations to performing activities of daily living without assistance, severe dysphagia, recurrent falls, or dementia. The questionnaire is relatively simple and easy, with high accuracy (72.3%), but in the validation study, it identified plenty of false positives (42.6%), potentially causing a large number of inappropriate referrals. Moreover, unlike MANAGE-PD, CDEPA does not screen for DAT eligibility or offer recommendations regarding treatment. The 5- (≥5 times oral levodopa tablet taken/day) 2- (≥2 h of OFF time/day) 1- (≥1 h of troublesome dyskinesia/day) criteria were proposed as screening criteria for inadequate symptom control based on the same Delphi expert consensus panel report that led to the development of MANAGE-PD [14,19,20,21]. 5-2-1 is easy to remember, and it can be used for quick and simple screening, but it evaluates only 3 out of 10 domains included in MANAGE-PD and does not offer further recommendations regarding subsequent treatment.

For the first time, data on the usability of the tool were collected in this study. The MANAGE-PD tool is easy and fast to fill in, which may explain why physicians and patients rated the tool’s usability as relatively high. However, physicians seemed to be a little less convinced about the benefit of the MANAGE-PD tool, as their rating on benefit was lower, with a mean score by physicians of 6.0 (on a scale from 1 to 10). The intentional simplicity of the tool may be a reason for the observation that the movement disorder specialists, i.e., physicians who may have the expertise and resources for comprehensive disease management, rated the benefit lower than the general neurologists. Thus, one may conclude that the tool is of more use to general neurologists. In the future, the usefulness of MANAGE-PD for movement disorder specialists might be enhanced with additional or more detailed questions [14]. However, this would increase the complexity of the tool, which might make it less attractive for general neurologists for whom the tool was originally designed [15]. It would be of future interest to further investigate the notable differences in concordance observed in our study between movement disorder specialists and general neurologists in the usage of MANAGE-PD.

### Limitations

This study has several shortcomings, many of which are inherent to all non-interventional studies. For instance, data may be skewed due to selection bias, as sites and patients participated based on convenience, and thus, only the subset of willing and motivated sites and patients were selected.

Moreover, this study was conducted exclusively in Germany. Thus, the generalizability of data for other countries can only be speculated on. To what extent national differences may be relevant for the usefulness of the tool and how concordance between physicians and the MANAGE-PD tool may be affected cannot be assessed in this study and may warrant further investigation. Studies investigating MANAGE-PD clinical utility under real-world conditions are currently ongoing in other countries and publications are expected in the future (J.K. personal communication). In addition, the population in this study does not necessarily reflect the German PD population. Since most sites (62.2%) were specialist clinics that usually manage more advanced patients, the patient population was somewhat skewed toward more severely affected patients. Thus, as the concordance with the tool differed depending on the type of institution, the results cannot be extrapolated to the total German population. Finally, a limitation of the MANAGE-PD tool itself is that it does not address levodopa-unresponsive patients, whose treatment requires clinical evaluation and judgment.

## 5. Conclusions

The MANAGE-PD tool proved again to be a valuable tool for neurologists to assess disease control and to identify patients who should be referred to specialist clinics for a DAT.

The fact that most PD patients were not sufficiently controlled according to the tool highlights the benefit of its use with respect to more objective and stringent patient management, especially for general neurologists.

The Parkinson Check self-assessment showed promising outcomes, which possibly merit its more widespread use.

The goal of reaching clear and unambiguous incremental treatment algorithms clearly benefits patients and physicians alike.

## Figures and Tables

**Table 1:** Patient characteristics.

	Disease Control Categorization by MANAGE-PD Tool				
	Category 1 (*n* = 50)	Category 2 (*n* = 125)	Category 3 (*n* = 103)	Total (*n* = 278)	*p*-Value
Age (years), mean ± SD	69.8 ± 7.6	71.3 ± 10.5	70.6 ± 10.8	70.8 ± 10.1	0.6693
Sex					
Male, *n* (%)	40 (80.0)	76 (60.8)	64 (62.1)	180 (64.7)	0.0438
Female, *n* (%)	10 (20.0)	49 (39.2)	39 (37.9)	98 (35.3)	
Time since diagnosis (years), mean ± SD	4.4 ± 4.1	7.5 ± 5.6	8.7 ± 5.6	7.4 ± 5.6	<0.0001
Time since first PD symptoms ^1^ (years), mean ± SD	6.5 ± 5.6	9.4 ± 5.8	10.0 ± 5.5	9.0 ± 5.8	0.003
Advanced stage of disease ^1^	13 (26.0)	84 (67.2)	83 (80.6)	180 (64.7)	<0.0001
Care-giver support, *n* (%)	3 (6.0)	56 (47.5)	61 (61.6)	120 (44.9)	<0.0001
Institution type, *n* (%)					
Specialist clinic ^2^	36 (72.0)	75 (60.0)	80 (77.7)	191 (68.7)	0.0142
Neurologist practice ^3^	14 (28.0)	50 (40.0)	23 (22.3)	87 (33.4)	

^1^ Physician’s judgment on the patient before implementing the MANAGE-PD tool. ^2^ Included university/specialist clinics and hospitals. ^3^ Included specialist or neurologist practices. Between-group comparisons, *p*-values were based on ANOVAs for numeric variables and Chi^2^ tests for categorical variables: Category 1, currently well controlled; Category 2, inadequately controlled—might benefit from further oral optimization; Category 3, inadequately controlled—might benefit from DAT; DAT, device-aided therapy; *n*, number of patients; SD, standard deviation.

**Table 2:** Tool acceptance by physicians and patients.

	Total		Specialist Clinic ^1^		Neurologist Practice ^2^	
	*n*	Mean ± SD	*n*	Mean ± SD	*n*	Mean ± SD
Physician						
Usability	18	8.1 ± 1.5	9	8.6 ± 0.9	9	7.7 ± 1.9
Benefit	18	6.0 ± 2.2	9	5.2 ± 2.5	9	6.8 ± 1.6
Time to complete MANAGE-PD tool Section 1 (mins)	278	6.1 ± 8.7	173	1.6 ± 3.4	87	13.3 ± 10.2
Time to complete MANAGE-PD tool Section 2 (mins)	278	5.9 ± 6.8	173	2.6 ± 4.7	87	10.7 ± 6.5
Patient						
Usability	263	7.0 ± 2.2	-	-	-	-
Time to complete MANAGE-PD tool Section 1 (mins) ^3^	275	6.1 ± 8.2	-	-	-	-

^1^ Includes university/specialist clinics and hospitals; one physician gave no information on usability/benefit. ^2^ Includes specialist/neurologist practices/other. ^3^ One implausible value was deleted: *n*, number of patients/physicians with valid data; SD, standard deviation.

## Data Availability

AbbVie is committed to responsible data sharing regarding the clinical trials we sponsor. This includes access to anonymized, individual, and trial-level data (analysis data sets), as well as other information (e.g., protocols, clinical study reports, or analysis plans), as long as the trials are not part of an ongoing or planned regulatory submission. This includes requests for clinical trial data for unlicensed products and indications. These clinical trial data can be requested by any qualified researchers who engage in rigorous, independent, scientific research and will be provided following review and approval of a research proposal, statistical analysis plan (SAP), and execution of a data sharing agreement (DSA). Data requests can be submitted at any time after approval in the US and Europe and after acceptance of this manuscript for publication. The data will be accessible for 12 months, with possible extensions considered. For more information on the process or to submit a request, visit the following link: https://vivli.org/ourmember/abbvie/ (accessed on 18 June 2024), then select “Home”.

## Supplementary Materials

The following supporting information can be downloaded at: https://www.mdpi.com/article/10.3390/brainsci14070630/s1, Figure S1: overview of MANAGE-PD Tool; Table S1: patients’ Response to MANAGE-PD Tool Section 1 by Category According to the MANAGE-PD tool.
