# Supplementary material for: Utilization of MANAGE-PD Tool in a Real-World Setting in Germany: A Cross-Sectional Study
Source: Brain Sci. 2024 Jun 24;14(7):630. doi: 10.3390/brainsci14070630 (PMC11275059; doi:10.3390/brainsci14070630)
Supplement: Supplementary file 1 [file brainsci-14-00630-s001.zip › brainsci-3022163-supplementary.pdf]

SUPPLEMENTAL MATERIAL

Utilization of MANAGE-PD Tool in a Real-World Setting in Germany: A Cross-Sectional Study

Martin Südmeyer [1,2,*], David J. Pedrosa [3], Frank Siebecker [4], Carolin Arlt [5], Jaakko J. Kopra [6] and Wolfgang H. Jost [7]

[1] Department of Neurology, Ernst-von-Bergmann Klinikum, 14467 Potsdam, Germany
[2] Department of Neurology, Medical Faculty, University Düsseldorf, 40225 Düsseldorf, Germany
[3] Department of Neurology, Philipps-University Marburg, 35037 Marburg, Germany; pedrosac@staff.uni-marburg.de
[4] Praxis Neurologie, 48291 Telgte, Germany; fs@neurologie-telgte.de
[5] AbbVie GmbH, 1230 Wien, Austria; carolin.arlt@abbvie.com
[6] AbbVie Deutschland GmbH & Co. KG, 65189 Wiesbaden, Germany; jaakko.kopra@abbvie.com
[7] Parkinson-Klinik Ortenau, 77709 Wolfach, Germany; w.jost@parkinson-klinik.de
* Correspondence: jeannette.bistri@klinikumevb.de; Tel.: +49-331-241-37102

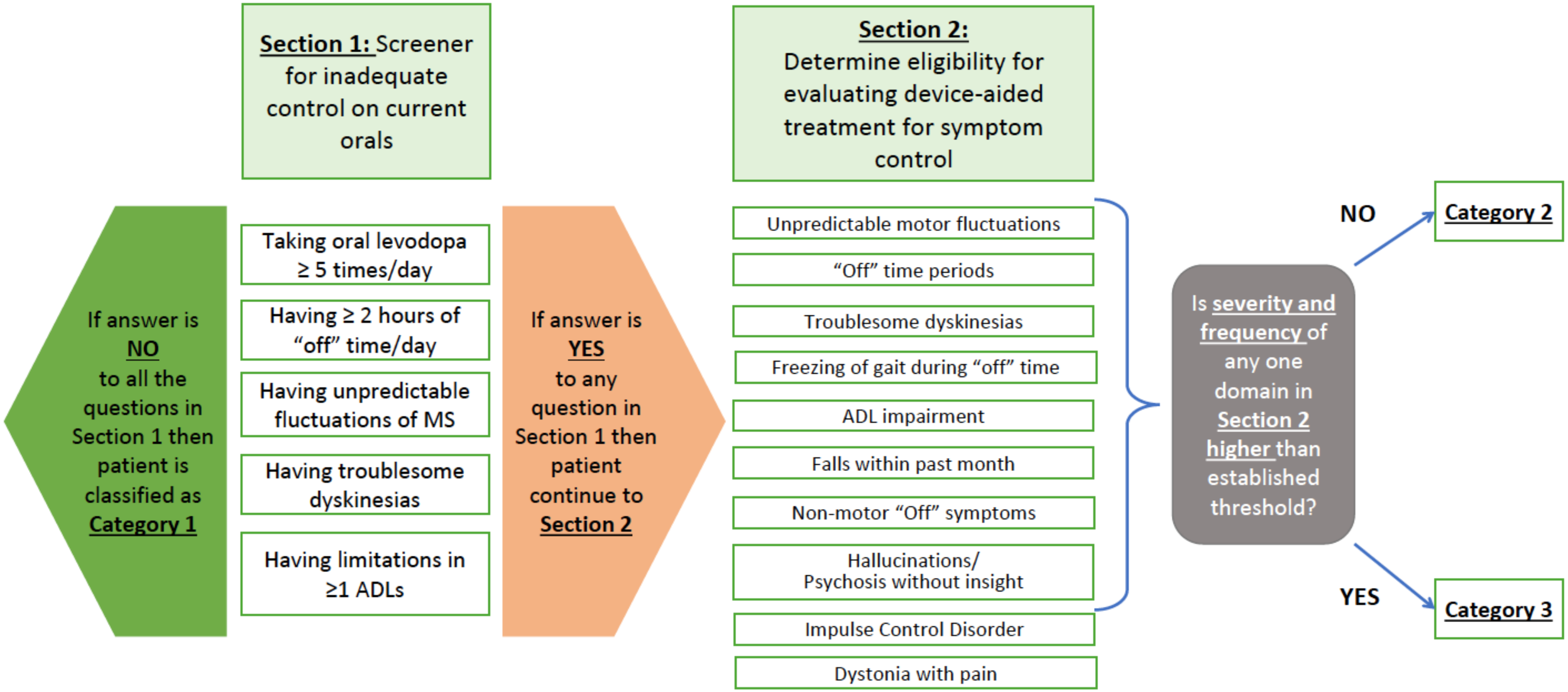

Figure S1. Overview of MANAGE-PD Tool.

Table S1. Patients' Response to MANAGE-PD Tool Section 1 by Category According to the MANAGE-PD tool.

	Disease Control by MANAGE-PD Tool			Total n (%)
	**Category 1 n (%)**	**Category 2 n (%)**	**Category 3 n (%)**	
Number of oral levodopa doses per day				
0-2 doses	4 (8.0)	8 (6.4)	0 (0.0)	12 (4.3)
3 doses	21 (42.0)	27 (21.6)	9 (8.7)	57 (20.5)
4 doses	23 (46.0)	25 (20.0)	25 (24.3)	73 (26.3)
≥ 5 doses	2 (4.0)	65 (52.0)	69 (67.0)	136 (48.9)
≥ 2 Hours of "Off" time per day	6 (12.0)	59 (47.2)	62 (60.2)	127 (45.7)
Unpredictable motor fluctuations	7 (14.0)	53 (42.4)	67 (65.0)	127 (45.7)
Troublesome dyskinesia	2 (4.0)	37 (29.6)	46 (44.7)	85 (30.6)
Limitations in ≥ 1 activities of daily living*	10 (20.0)	92 (74.2)	85 (82.5)	187 (67.5)

*The information was missing for one patient in Category 2.

Category 1, currently well controlled; Category 2, inadequately controlled – might benefit from further oral optimization; Category 3, inadequately controlled – might benefit from DAT; DAT, device-aided therapy; n, number of patients.
